# Evidence of recombination in quasispecies populations of a Hepatitis C Virus patient undergoing anti-viral therapy

**DOI:** 10.1186/1743-422X-3-87

**Published:** 2006-10-24

**Authors:** María P Moreno, Didier Casane, Lilia López, Juan Cristina

**Affiliations:** 1 Laboratorio de Virología Molecular, Centro de Investigaciones Nucleares, Facultad de Ciencias, Universidad de la República, Iguá 4225, 11400 Montevideo, Uruguay; 2 Laboratoire Evolution Génomes et Spéciation (UPR9034), CNRS, 91198 Gif-sur-Yvette, France

## Abstract

**Background/Aim:**

Hepatitis C virus (HCV) has been the subject of intense research and clinical investigation as its major role in human disease has emerged. HCV circulates *in vivo *as a complex population of different but closely related viral variants, commonly referred to as a quasispecies. The extent to which recombination plays a role in the evolution of HCV quasispecies when patients are undergoing anti-viral therapy is currently unknown. In order to gain insight into these matters, we have performed a phylogenetic analysis of HCV quasispecies populations from six patients undergoing anti-viral therapy.

**Methods:**

Putative recombinant sequences were identified with the use of SimPlot program. Recombination events were confirmed by bootscaning, using putative recombinant sequence as a query. Statistical support for the presence of a recombination event was done by the use of LARD program.

**Results:**

A crossing-over event in the NS5A gene in a HCV strain recovered after four weeks of treatment was identified in quasispecies from a patient with sustained response. Putative parental-like strains were identified as strains circulating in previous weeks on the same patient.

**Conclusion:**

Only one recombinant strain was detected in all patient quasispecies populations studied. The recombination break-point is situated on the PKR-binding region of NS5A. Although recombination may not appeared to be extensive in NS5A genes of HCV quasispecies populations of patients undergoing antiviral therapy, this possibility should be taken into account as a mechanism of genetic variation for HCV.

## Background

Hepatitis C virus (HCV) is estimated to infect 170 million people worldwide and creates a huge disease burden from chronic, progressive liver disease [1] HCV has become a major cause of liver cancer and one of the commonest indications of liver transplantation [2,3].

HCV has been classified in the family *Flaviviridae*, although it differs from other members of the family in many details of its genome organization [1]. Like most RNA viruses, HCV circulates *in vivo *as a complex population of different but closely related viral variants, commonly referred to as a quasispecies [4-7].

HCV is an enveloped virus with an RNA genome of approximately 9400 bp in length [8,9]. Comparison of nucleotide sequences of variants recovered from different individuals and geographical regions has revealed the existence of at least six major genetic groups [1,10-12]. Each of the six major genetic groups of HCV contains a series of more closely related sub-types [1].

Interferon monotherapy provided the first hope for patients with chronic hepatitis C that the virus could be permanently eradicated. An important development in treating this disease was the recognition that the effects of interferon could be greatly enhanced by combining it with ribavirin, a nucleoside analogue. This combination regimen essentially doubled the sustained virological response rates seen with interferon alone. Recently, pegylated forms of interferon have been developed, and when that forms of interferon are used in combination with ribavirin, it demonstrates even better efficacy. For that reason, peginterferon alfa-2a and peginterferon alfa-2b are the latest innovations for the treatment of chronic hepatitis C [3,13,14].

Recombination plays a significant role in the evolution of RNA viruses by creating genetic variation. For example, the frequent recovery of poliovirus that results from recombination has the potential to produce "escape mutants" in nature as well as in experiments [15]. Recombination has also been detected in other RNA viruses for which multivalent vaccines are in use or in trials [16,17].

Recently, a natural intergenotypic recombinant (2 k/1b) of HCV has been identified in Saint Petersburg (Russia) [18,19]. Phylogenetic analyses of HCV strains circulating in Peru, demonstrated the existence of natural intra-genotypic HCV recombinant strains (1a/1b) circulating in the Peruvian population [20]. In these cases, the recombination events have taken place in the non-structural region of the HCV genome. Recombination break-points in HCV structural capsid genomic region has been recently identified [21].

Given the implications that recombination has for RNA virus evolution [16], it is clearly important to determine the extent to which recombination plays a role in the evolution of HCV quasispecies populations *in vivo*, when patients are undergoing anti-viral therapy.

## Results

**Figure 1:**
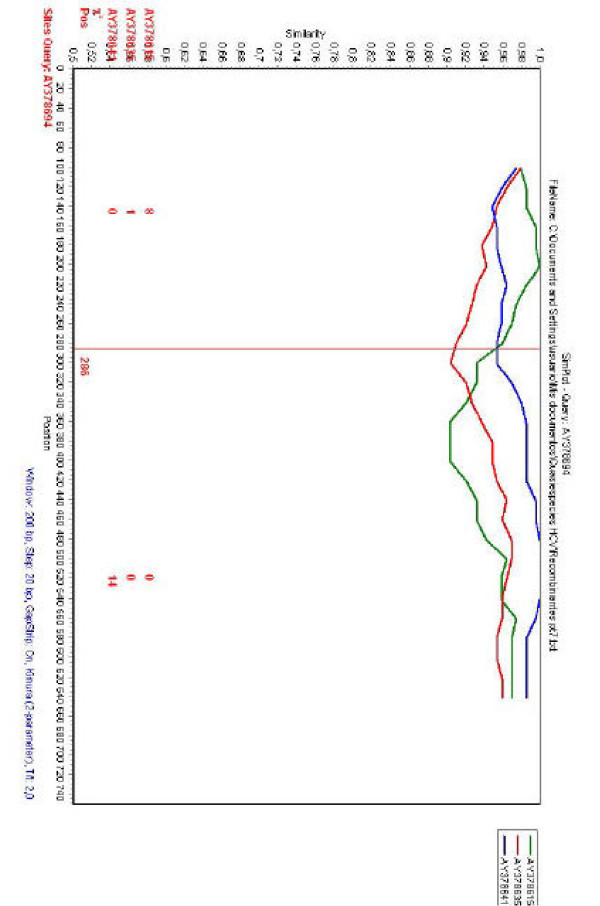
**Phylogenetic profiles of HCV sequences**. Results from SimPlot analysis are shown. The query sequence (AY378694) is indicated on the upper part of the figure. Sequences to be compared with the query sequence are indicated on the right side of the figure. When comparisons were done, SimPlot generates a similarity plot using the Kimura-two parameter distance model in a sliding window of 200 nucleotides, moving 20 nucleotides between plots. The *y*-axis gives the percentage of identity found. Comparison of HCV strain AY378694 with strains AY378615, AY378641 and AY378635 is shown. The red vertical line shows the recombination point at position 286. Red numbers on the bottom part of the figure denote the number of informative sites that support clustering of the query sequence with the respective strains indicated in red on the bottom left side of the figure.

To gain insight into possible recombination events, a phylogenetic profile analysis was carried out using HCV NS5A sequences from HCV quasispecies populations obtained by Puig-Basgoiti *et al*. [22] from patients undergoing anti-viral therapy. Sequences were obtained by the use of the LANL database [23] (for patients, strains accession numbers, quasispecies obtained at different time points during therapy and therapy outcome, see Table 1). Phylogenetic profile analysis was done by the use of the SimPlot program [24]. Interesting, when the analysis was carried out for strain AY378694 (obtained on week 4 in patient No. 7, see Table 1), a recombination point was detected at position 286 of the NS5A sequence alignment and two putative parental-like strains (AY378615 and AY378641, obtained on weeks 0 and 2 from the same patient, respectively) were identified (see Fig. 1 and Table 1).

**Table 1:** Quasispecies population in HCV patients undergoing anti-viral therapy^a^

**Patient ID**	**Weeks on therapy**	**Strains in population^b^**	**Treatment response^c^**
PAT 7	0	AY378615 to AY378634	SR
	1	AY378635 to AY378650	SR
	2	AY378651 to AY378673	SR
	4	AY378674 to AY378694	SR
			
PAT 8	0	AY378705 to AY378729	SR
	1	AY378730 to AY378750	SR
	4	AY378751 to AY378775	SR
			
PAT 9	0	AY378776 to AY378799	SR
	1	AY378800 to AY378821	SR
	2	AY378822 to AY378869	SR
	4	AY378846 to AY378869	SR
			
PAT 10	0	AY381300 to AY381324	NR
	1	AY381325 to AY381344	NR
	2	AY381345 to AY381369	NR
	4	AY381370 to AY381394	NR
			
PAT11	0	AY381414 to AY381434	NR
	1	AY381435 to AY381455	NR
	2	AY381456 to AY381480	NR
	4	AY381481 to AY381505	NR
			
PAT12	0	AY381528 to AY381549	NR
	1	AY381550 to AY381570	NR
	2	AY381571 to AY381595	NR
	4	AY381596 to AY381619	NR

^a^According to Puig-Basagoity *et al*. [22].

^b^Strains in quasispecies populations are indicated by accession numbers.

^c^SR means sustained response, NR means no response.

**Figure 2:**
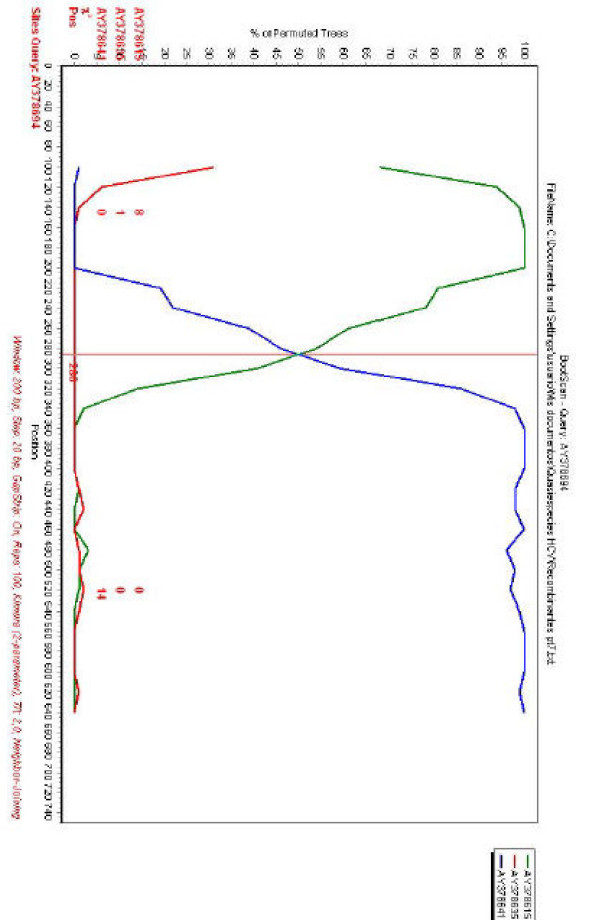
**Bootscanning of HCV sequences**. Query sequence (AY378694) is shown on the upper part of the figure. Sequences to be compared with the query sequence are indicated on the right side of the figure. When comparisons were done, SimPlot generates a graph of percentage of permutated trees obtained using a sliding window of 200 nucleotides, moving 20 nucleotides at a time. The *y*-axis gives the percentage of permutated trees. This approach permits to observe levels of phylogenetic relatedness between a query sequence and a reference sequence in different genomic regions. The rest same as Fig. 1A.

In order to confirm these results, the same sequences were used for a bootscanning study [25]. The basic principle of bootscanning is that mosaicism is suggested when one observes high levels of phylogenetic relatedness between a query sequence and more than one reference sequence [25].When the putative recombinant strain identified in the previous analysis (AY378694) is used as a query, this is observed for this strain and the two putative parental-like strains previously detected (see Fig. 2). The same recombination break-point position is observed in the bootscanning analysis (see Figs. 1 and 2), confirming a recombination break-point at position 286 of the NS5A of strain AY378694.

**Figure 3:**
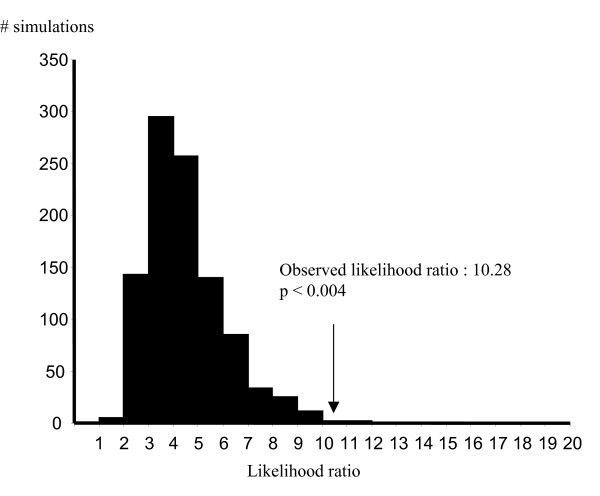
**Distribution of the likelihood ratios expected by chance**. The distribution of likelihood ratios for the null hypothesis (i.e. no recombination) is shown. The *y*-axis shows the number of simulations. Likelihood ratios are shown at the bottom of the figure. The arrow shows the likelihood ratio obtained for the real dataset for the putative recombinant strain.

To assess whether the recombination model we obtained gave a significantly better fit to the data than the null hypothesis of no recombination, we used LARD [26]. The results of these studies are shown in Fig. 3.

As it can be seen in the figure, simulations of sequence evolution under the null hypothesis (i.e., no recombination) gave strong statistical support for the alternative hypothesis of recombination (*P *< 0.004, Fig. 3).

## Discussion

The extent to which recombination plays a role in the evolution of HCV quasispecies when patients are undergoing anti-viral therapy is currently unknown. In order to get insight into this issue, we performed phylogenetic studies on the NS5A gene of HCV quasispecies populations from patients undergoing antiviral therapy. We have selected NS5A since previous work by Enomoto *et al*. [27] suggested that the genetic heterogeneity of a specific domain of HCV NS5A, termed IFN sensitivity-determining region (ISDR), was related closely to response in Japanese patients. Although this issue continues to be controversial [22], analysis of the published information supports the hypothesis that a relationship exists between NS5A and response to therapy [28,29].

**Figure 4:**
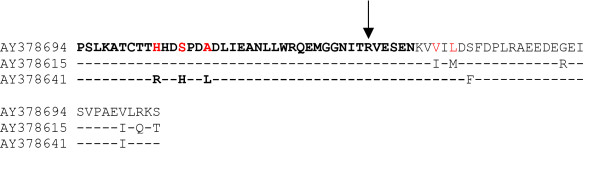
**Alignment of amino acid sequences of the PKR-binding region and ISDR of recombinant and putative parental-like strains**. Strains are indicated by accession number in the left side of the figure. Identity to strain recombinant strain AY378694 in putative parental-like strains AY378615 and AY 378641 is shown by a dash. PKR-binding region and ISDR sequences are shown in bold. Recombination break-point is indicated by an arrow. Amino acids known to be present in interferon resistant viruses are indicated in red in recombinant strain AY378694 (see refs. [31, 35]).

The results of these studies reveal that recombination can not be denied as an evolutionary mechanism for generating diversity in HCV *in vivo*, in patients undergoing anti-viral therapy (see Figs. 1 and 2). Recombination does not seems to play an extensive roll in the evolution of HCV quasispecies populations, at least by the study of NS5A genes, since only one recombinant isolate was observed among all HCV quasispecies populations studied. On the other hand, the true frequency of recombination may be underestimated, since a statistical significant number of differences among recombinant and putative parental-like strains are needed in order to achieve detection by current methods applied to detect recombination events. Recombination may serve two opposite purposes: exploration of a new combination of genomic region from different origins or rescuing of viable genomes from debilitated parental genomes [30]. Interestingly, the recombination break-point identified in recombinant strain AY378694 is situated on the PKR-binding region of NS5A ISDR [27,31] (see Fig. 4). Recent evidence suggests that HCV NS5A protein can repress PKR function *in vivo*, possibly allowing HCV to escape the antiviral effects of interferon [1,3,32,33]. An analysis of NS5A translated sequences of recombinant and putative parental-like virus suggests the possibility that recombinant isolate AY378694 may have acquired amino acids known to be present in HCV strains resistant to interferon treatment (see Fig. 4), although more work is needed in order to test this hypothesis, since the results found for the ISDR still remains a controversial issue [22]. For that reason, although recombination may not appeared to be extensive in NS5A genes of HCV quasispecies populations of patients undergoing antiviral therapy, this possibility should be taken into account as a mechanism of genetic variation for HCV.

## Conclusion

Only one recombinant strain was detected in all patient quasispecies populations studied. The recombination break-point identified in strain AY378694 is situated on the PKR-binding region of NS5A. The results of these studies reveal that recombination events can be observed in patients undergoing anti-viral therapy. Recombination can not be denied as a mechanism of genetic variation for HCV.

## Methods

### Patients and strains

Patients and strains refereed in these studies belong to a recent study done by Puig-Basagoiti *et al*. [22]. All patients had genotype 1b infection. Treatment consisted of the administration of IFN-α-2b and ribavirin (see ref. [22]).

### Sequences

NS5A sequences from the study done by Puig-Basagoity *et al*. [22] were obtained from the HCV LANL database [23] (for patient identification, quasispecies accession numbers obtained at each week of therapy and therapy outcome, see Table 1). Sequences were aligned using the CLUSTAL W program [34].

### Recombination analysis

Putative recombinant sequences were identified with the SimPlot program [24]. This program is based on a sliding window method and constitutes a way of graphically displaying the coherence of the sequence relationship over the entire length of a set of aligned homologous sequences. The window width and the step size were set to 200 bp and 20 bp, respectively.

### Bootscanning analysis

The results obtained in the recombination analysis were confirmed using a bootscanning analysis [25]. The window width and the step size were set to 200 bp and 20 pb, respectively.

### LARD analysis

To assess whether the recombination model we obtained gave a significantly better fit to the data than the null hypothesis of no recombination, we used LARD [26]. Briefly, for every possible breakpoint, the sequence alignment was divided into two independent regions for which the branch lengths of a tree of the putative recombinant and its two parent sequences were optimised. The two results (likelihoods) obtained by using the separate regions were then combined to give a likelihood score for that breakpoint position and the breakpoint position that yielded the highest likelihood then was compared, by using a likelihood ratio test, to the likelihood obtained from the same data under a model that permitted no recombination. The likelihood ratio obtained by using the real data were evaluated for significance against a null distribution of likelihood ratios produced by using Monte Carlo simulation of sequences generated without recombination. Sequences were simulated 1,000 times by using the maximum likelihood model parameters and sequence lengths from the real data using Seq-Gen [35].

## Competing interests

The author(s) declare that they have no competing interests.

## Authors' contributions

JC and PM conceived the study. JC, PM and LL designed the analysis. JC and PM performed the SimPlot and Bootscanning analysis. DC performed the LARD analysis and contributed to the discussion of all results found in this work. JC wrote the paper.

## References

[B1] Simmonds P (2004). Genetic diversity and evolution of hepatitis C virus 15 years on. J Gen Virol.

[B2] Hoofnagle JH (2003). Course and outcome of hepatitis C. Hepatology.

[B3] Pawlotsky JM (2003). The nature of interferon-alfa resistance in hepatitis C virus infection. Curr Opin Infect Dis.

[B4] Chambers TJ, Fan X, Droll DA, Hembrador E, Slater T, Nickells MW, Dustin LB, Dibisceglie AM (2005). Quasispecies heterogeneity within the E1/E2 region as a pretreatment variable during pegylated interferon therapy of chronic hepatitis C virus infection. J Virol.

[B5] Laskus T, Wilkinson J, Gallegos-Orozco JF, Radkowski M, Adair DM, Nowicki M, Operskalsi E, Buskell Z, Seeff LB, Vargas H, Rakela J (2004). Analysis of hepatitis C virus quasispecies transmission and evolution in patients infected through blood transfusion. Gastroenterology.

[B6] Feliu A, Gay E, Garcia-Retortillo M, Saiz JC, Foms X (2004). Evolution of hepatitis C virus quasispecies immediately following liver transplantation. Liver Transpl.

[B7] Martell M, Esteban JL, Quer J, Genesca J, Weiner A, Esteban R, Guardia J, Gomez J (1992). Hepatitis C virus (HCV) circulates as a population of different but closely related genomes: quasispecies nature of HCV genome distribution. J Virol.

[B8] You S, Stump DD, Branch AD, Rice CM (2004). A *cis*-actingreplication element in the sequence encoding the NS5B RNA-dependent RNA polymerase is required for hepatitis C virus RNA replication. J Virol.

[B9] Pestova TV, Shatsky IN, Fletcher SP, Jackson RJ, Hellen CUT (1998). A prokaryotic-like mode of cytoplasmic eukaryotic ribosome binging to the initiation codon during internal translation initiation of hepatitis C and classical swine fever virus RNAs. Genes Dev.

[B10] Simmonds P, Bukh J, Combet C, Deleage G, Enomoto N, Feinstone S, Halfon P, Inchauspe G, Kuiken C, Maertens G, Mizokami M, Murphy DG, Okamoto H, Pawlotsky JM, Penin F, Sablon E, Shin IT, Stuyver LJ, Thiel HJ, Viazov S, Weiner AJ, Widell A (2005). Consensus proposals for a unified system of nomenclature of hepatitis C virus genotypes. Hematology.

[B11] Simmonds P (2001). The origin and evolution of hepatitis viruses in humans. J Gen Virol.

[B12] Simmonds P, Holmes EC, Cha TA, Chan SW, McOmish F, Irvine B, Beall E, Yap PL, Kolberg J, Urdea MS (1993). Classification of hepatitis C virus into six major genotypes and a series of subtypes by phylogenetic analysis of the NS-5 region. J Gen Virol.

[B13] Zeuzem S (2004). Heterogenous virologic response rates to interferon-based therapy in patients with chronic hepatitis C: who responds less well?. Ann Intern Med.

[B14] Fried MW, Hadziyannis SJ (2004). Treatment of chronic hepatitis C infection with peginterferons plus ribavirin. Semin Liver Dis.

[B15] Kew OM, Nottay BK, Chanock RM, Lerner RA (1984). Evolution of the oral polio vaccine strains in humans occurs by both mutation and intra-molecular recombination. Modern Approaches to Vaccines.

[B16] Worobey M, Holmes EC (1999). Evolutionary aspects of recombination in RNA viruses. J Gen Virol.

[B17] Suzuki Y, Gojobori T, Nakagomi O (1998). Intragenic recombinations in rotaviruses. FEBS Lett.

[B18] Kalinina O, Norder H, Magnius O (2004). Full-length open reading frame of a recombinant hepatitis C virus strain from St. Petersburg: proposed mechanism of its formation. J Gen Virol.

[B19] Kalinina O, Norder H, Mukomolov S, Magnius LO (2002). A natural intergenotypic recombinant of hepatitis C virus identified in St. Petersburg. J Virol.

[B20] Colina R, Casane D, Vasquez S, Garcia-Aguirre L, Chunga A, Romero H, Khan B, Cristina J (2004). Evidence of intratypic recombination in natural populations of hepatitis C virus. J Gen Virol.

[B21] Cristina J, Colina R (2006). Evidence of structural genomic region recombination in Hepatitis C virus. Virology J.

[B22] Puig-Basagoiti F, Forns X, Furcic I, Ampurdanes S, Gimenez-Barcons M, Franco S, Sanchez-Tapias JM, Saiz JC (2005). Dynamics of hepatitis C virus NS5A quasispecies during interferon and ribavirin therapy in responder and non-responder patients with genotype 1b chonic hepatitis C. J Gen Virol.

[B23] Kuiken C, Yusim K, Boykin L, Richardon R (2005). The HCV Sequence Database. Bioinformatics.

[B24] Lole KS, Bollinger RC, Parnjape RS, Gadkari D, Kulkarni SS (1999). Full-length human immunodeficiency virus type 1 genomes from subtype C-infected seroconverters in India, with evidence of intersubtype recombination. J Virol.

[B25] Salminen MO, Carr JK, Burke DS, McCutchan FE (1995). Identification of breakpoints in intergenotypic recombinants of HIV type 1 by bootscanning. AIDS Res Hum Retroviruses.

[B26] Holmes EC, Worobey M, Rambaut A (1999). Phylogenetic evidence for recombination in dengue virus. Mol Biol Evol.

[B27] Enomoto N, Sakuma I, Asahina Y, Kurosaki M, Murakami T, Yamamoto C, Izumi N, Marumo F, Sato C (1995). Comparison of full-length sequences of interferon-sensitive and resistant hepatitis C virus 1b. Sensitivity to interferon is conferred by amino acid substitutions in the NS5A gene. J Clin Investig.

[B28] Gimenez-Barcons M, Franco S, Suarez Y, Forns X, Ampurdanes S, Puig-Basagoiti F, Sanchez-Fueyo A, Barrera JM, Llovet JM, Bruix J, Sanchez-Tapias JM, Rodes J, Saiz JC (2001). High amino acid variability within the NS5A of hepatitis C virus (HCV) is associated with hepatocellular carcinoma in patients with HCV-1b-related cirrhosis. Hepatology.

[B29] Witherell GW, Beineke P (2001). Statistical analysis of combined substitutions in non-structural 5A region of hepatitis C virus and interferon response. J Med Virol.

[B30] Domingo E, Holland JJ (1997). RNA virus mutations and fitness for survival. Annu Rev Microbiol.

[B31] Gale MJ, Korth MJ, Tang NM, Tan SL, Hopkins DA, Dever TE, Polyak SJ, Gretch DR, Katze MG (1997). Evidence that hepatitis C virus resistance to interferon is mediated through repression of the PKR protein kinase by the nonstructural 5A protein. Virology.

[B32] Gale MJ, Blakely CM, Kwieciszewski B, Tan SL, Dossett M, Tang NM, Korth MJ, Polyak SJ, Gretch DR, Katze MG (1998). Control of PKR protein kinase by Hepatitis C Virus nonstructural 5A protein: molecular mechanisms of kinase regulation. Mol Cel Biol.

[B33] Pawlotsky JM, Germanidis G, Neumann AU, Pellerin M, Frainais PO, Dhumeaux D (1998). Interferon Resistance of Hepatitis C Virus genotype 1b: relationship to nonstructural 5A gene quasispecies mutations. J Virol.

[B34] Thompson JD, Higgins DG, Gibson TJ (1994). CLUSTAL W: improving the sensitivity of progressive multiple sequence alignment through sequence weighting, position-specific gap penalties and weight matrix choice. Nucleic Acid Res.

[B35] Rambaut A, Grassly NC (1997). Seq-Gen: an application for the Monte Carlo simulation of DNA sequence evolution along phylogenetic trees. Comp Appl Biosci.

[B36] Enomoto N, Sakuma I, Asahina Y, Kurosaki M, Murakami T, Yamamoto C, Ogura Y, Izumi N, Maruno F, Sato C (1996). Mutations in the non-structural protein 5A gene and response to interferon in patients with chronic hepatitis C virus 1b infection. N Engl J Med.

[B37] Pawlotsky JM, Germanidis G, Neumann AU, Pellerin M, Frainais PO, Dhumeaux D (1998). Interferon Resistance of Hepatitis C Virus genotype 1b: relationship to nonstructural 5A gene quasispecies mutations. J Virol.

